# *Thyrotropin-Releasing Hormone* Gene Methylation as a Potential Biomarker for Anal Intraepithelial Neoplasia

**DOI:** 10.3390/ijms262411784

**Published:** 2025-12-05

**Authors:** Chavis Pholpong, Nittaya Phanuphak, Tippawan Pankam, Supranee Buranapraditkun, Nakarin Kitkumthorn, Bernett Lee, Parvapan Bhattarakosol, Arkom Chaiwongkot

**Affiliations:** 1 Interdisciplinary Program of Biomedical Sciences, Graduate School, Chulalongkorn University, Bangkok 10330, Thailand; 6681009520@student.chula.ac.th; 2 Center of Excellence in Applied Medical Virology, Faculty of Medicine, Chulalongkorn University, Bangkok 10330, Thailand; parvapan.b@chula.ac.th; 3 Institute of HIV Research and Innovation, Bangkok 10330, Thailand; nittaya.p@trcarc.org; 4 Department of Medical Technology, College of Allied Health Sciences, Rattana Bundit University, Pathum Thani 12160, Thailand; pankamt@yahoo.com; 5 The Thai Red Cross AIDS Research Centre, Bangkok 10330, Thailand; 6 Division of Allergy and Clinical Immunology, Department of Medicine, King Chulalongkorn Memorial Hospital, Faculty of Medicine, Chulalongkorn University, Thai Red Cross Society, Bangkok 10330, Thailand; bsuprane2001@yahoo.com; 7 Center of Excellence in Thai Pediatric Gastroenterology, Hepatology and Immunology (TPGHAI), Faculty of Medicine, Chulalongkorn University, Bangkok 10330, Thailand; 8 Center of Excellence in Vaccine Research and Development (Chula Vaccine Research Center—Chula VRC), Faculty of Medicine, Chulalongkorn University, Bangkok 10330, Thailand; 9 Faculty of Dentistry, Burapha University, Chonburi 20131, Thailand; nakarinkit@gmail.com; 10 Lee Kong Chian School of Medicine, Nanyang Technological University, Experimental Medicine Building, 59 Nanyang Drive, Singapore 636921, Singapore; bernett.lee@ntu.edu.sg; 11 Department of Microbiology, Faculty of Medicine, Chulalongkorn University, Bangkok 10330, Thailand

**Keywords:** *thyrotropin-releasing hormone (TRH)*, methylation, anal intraepithelial neoplasia, anal cancer screening, biomarker, men who have sex with men (MSM), human papillomavirus

## Abstract

Anal cancer is high in men who have sex with men living with human immunodeficiency virus (MSM-LWHIV). This cancer is strongly associated with high-risk human papillomavirus (HR-HPV) infection. Anal cancer screening using cytology and high-resolution anoscopy (HRA) for diagnosis of anal intraepithelial neoplasia requires specialized expertise. Biomarkers for the diagnosis of abnormal anal cells are of interest. *Thyrotropin-releasing hormone (TRH)* methylation at cg01009664 was detected using a pyrosequencing assay to compare methylation patterns among different anal lesions. Our results demonstrated that *TRH* methylation was significantly hypermethylated in anal intraepithelial neoplasia (AIN3) (>20%) and AIN1-2 (>10%) but less methylated in normal (<10%) (*p* < 0.001). *TRH* gene methylation showed higher sensitivity than the cytology for predicting AIN1+ (75.96% vs. 25.37%, respectively) and AIN2+ (78.95%% vs. 19.23%, respectively). There was no significant correlation between *TRH* methylation and the percentage of CD4 in patients with HIV (*p* > 0.05). *TRH* methylation in anal swabs reflects the presence of anal intraepithelial neoplasia. Methylation analysis showed higher sensitivity than cytology for high-grade lesions and was independent of immune status. These findings support its use as a screening tool to preselect patients for HRA, potentially reducing unnecessary procedures while maintaining diagnostic accuracy.

## 1. Introduction

Anal cancer, although relatively rare, is of significant concern due to its increasing incidence among men who have sex with men living with human immunodeficiency virus (MSM-LWHIV), women with HIV, MSM without HIV, women with a history of cervical/vaginal/vulva cancers and precancers, and non-HIV immunosuppressed people such as solid organ transplant recipients (SOTRs) [1,2,3,4]. This cancer is strongly associated with high-risk human papillomavirus (HR-HPV) infection, particularly HPV16 [5,6], which plays a crucial role in its pathogenesis. People in an immunocompromised state, such as those with primary immunodeficiencies (PIDs), solid organ transplant recipients of immunosuppression and acquired immunodeficiency, e.g., HIV infection, are particularly vulnerable for HPV-related diseases, with incidence rates markedly higher compared to the general population [7,8]. Anal cancer screening can be detected by anal cytology and high-resolution anoscopy (HRA) with directed biopsy for histology examination. Anal cytology is diagnosed with different lesions, including negative for intraepithelial lesion or malignancy (NILM), low-grade squamous intraepithelial lesion (LSIL), atypical squamous cells of undetermined significance (ASC-US), atypical squamous cells cannot exclude high grade (ASC-H), high-grade squamous intraepithelial lesion (HSIL), and anal squamous cell carcinoma (ASCC) [9]. Anal cytology and HR-HPV tests have good sensitivity but limited specificity [2,9]. The sensitivity of anal cytology for detecting AIN ranges from 47% to 90%, with higher sensitivity observed in more advanced lesions. However, its specificity is lower, ranging from 32% to 60% [10,11,12]. HRA is still the gold-standard diagnostic method for anal intraepithelial neoplasia (AIN), but its use is limited due to the need for specific training and resources [13].

The prevalence of AIN2+ was the highest among MSM-LWHIV and persons living with HIV (PLWH) [14]. It has been reported that treating anal precancerous HSIL significantly reduces risk of progression to anal cancer among PLWH [15]. The current anal cancer screening depends on cytological and histological examinations which require specialized expertise. Biomarkers for abnormal anal cell diagnosis are of interest.

Previous studies have shown that hypermethylation of *thyrotropin-releasing hormone (TRH)* gene was detected in oral and oropharyngeal cancer [16]. It can also be used to identify high-grade cervical lesions or cervical intraepithelial neoplasia (CIN2+) [17]. It would be interesting to detect *TRH* gene methylation in other HPV-related cancers. The *TRH* gene is located on chromosome 3 and is a tripeptide hormone (pGlu-His-Pro-NH_2_) that is mainly produced in the hypothalamus and stimulates the synthesis and release of Thyroid Stimulating Hormone (TSH) as well as the synthesis of prolactin (PRL). It is crucial for controlling metabolism, energy balance, and hormone secretion. It is also found in other peripheral tissues, including the cervix [18]. Beyond its endocrine functions, *TRH* has been linked to cancer development and progression. *TRH* has been found as a potential biomarker in breast cancer [18], acute myeloid leukemia (AML) [19], and melanoma [20].

Initially, the GSE186859 dataset of anal samples [21] retrieved from NCBI was analyzed and it was found that the *TRH* gene at cg01009664 was hypermethylated in AIN3/anal cancer samples compared to normal cells.

Therefore, we further validated this finding in clinical anal samples with different lesion severity using a pyrosequencing assay to detect the *TRH* methylation at cg01009664. The diagnostic performance of the assay was analyzed.

## 2. Results

### 2.1. Analysis of GSE186859 Dataset and TRH Methylation in Cervical Cell Lines

**Figure 1:**
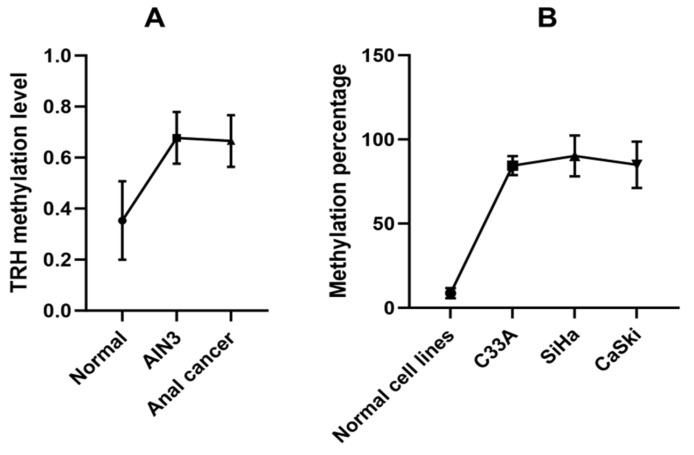
*TRH* methylation analysis. (**A**) Data obtained from GSE186859 dataset, methylation levels at cg01009664 of *TRH* gene in anal cells with different anal lesions stratified by histology as normal (9 samples), AIN3 (13 samples) and anal cancer (121 samples). (**B**) Pyrosequencing analysis data of normal and cervical cancer cell lines.

The GSE186859 dataset of methylation comprised over 485,513 CpG positions was downloaded from the Gene Expression Omnibus (GEO) datasets. The dataset includes samples from 9 normal, 13 AIN3, and 121 anal cancer cases. The GEO2R tool was used for differential methylation analysis between normal and AIN3/CA samples and log2FC ≥ 0.3 was used as cut-off to identify highly methylated CpG positions. There were 2088 CpG positions (t > 3 and *p* < 0.05), representing 537 genes, which are listed in Supplementary Table S1. The STRING database was used to analyze gene ontology (GO) and KEGG pathway analysis. The significant KEGG pathways included neuroactive ligand–receptor interaction and cAMP signaling pathway. The biological processes (BPs) included nervous system development, system development, multicellular organism development and cell–cell signaling. The molecular functions (MFs) included DNA-binding transcription factor activity, RNA polymerase II transcription regulatory region sequence-specific DNA binding and transcription regulator activity. Cellular components (CCs) predominantly involved synaptic membrane, cell junction and intrinsic component of plasma membrane. All GO and KEGG pathway of 537 genes are listed in Supplementary Table S2. *TRH*, which is involved in neuroactive ligand–receptor interaction, is primarily found in the cell periphery and plasma membrane. Its functions include binding, receptor–ligand activity, and signaling receptor regulator activity. It is also involved in biological processes such as cell–cell signaling, multicellular organismal processes, and cellular process regulation. Our group has previously shown that cg01009664 position of *TRH* gene was substantially methylated in cervical cancer cases when compared to normal cervical cells [17]. The cg01009664 position in the anal sample GSE186859 dataset was analyzed and we found that it was markedly methylated in ≥AIN3 cells when compared to normal anal cells as shown in Figure 1A.

**Figure 2:**
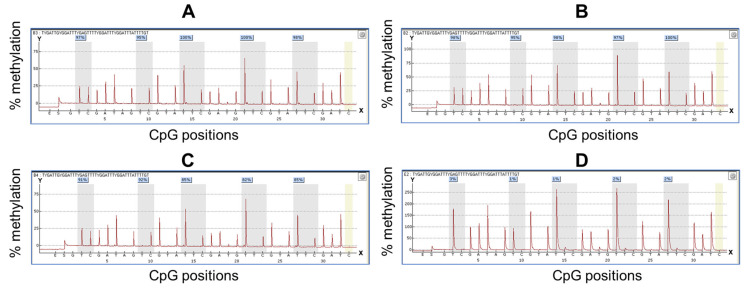
Pyrogram from *TRH* methylation quantification by pyrosequencing. *Y*-axis represents percentage of methylation, *X*-axis represents CpG positions. The yellow bar represents the unmethylated cytosine control within the analyzed sequence. The methylation percentage values of each CpG site were shown in the box on the top of the gray bar. (**A**–**D**) are pyrogram results obtained from four cell lines including (**A**) CaSki, (**B**) SiHa, (**C**) C33A and (**D**) normal cervical cell lines PCS480011.

To analyze TRH gene methylation at the cg01009664 position using the pyrosequencing assay, we first tested the assay in normal and cervical cancer cell lines, which showed low and high TRH methylation levels, respectively, as shown in Figure 1B. Pyrogram of five CpGs of CaSki, SiHa and C33A cervical cancer cell lines showed high methylation levels (80–100%) while normal cervical cell lines revealed low methylation levels (<10%) as shown in Figure 2.

### 2.2. Analysis of TRH Methylation in Anal Samples

**Figure 3:**
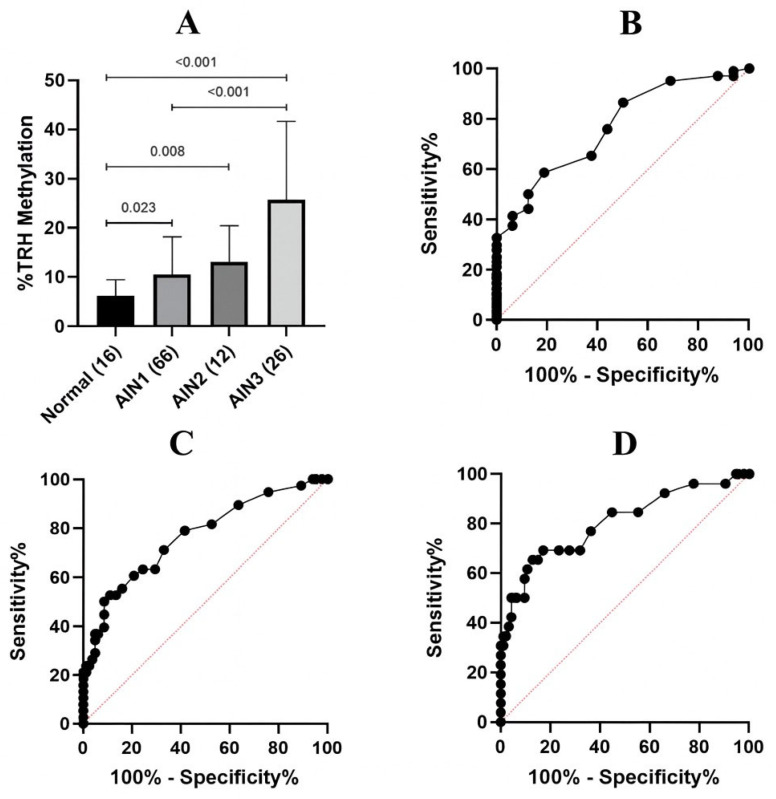
*TRH* methylation analysis in anal samples with different lesion severity. (**A**) Average methylation percentage of five CpGs at cg01009664 of *TRH* gene in anal samples stratified by histology as normal, AIN1, AIN2 and AIN3. Error bars in the bar graph represent the mean with standard deviation (SD). There were statistically significant differences among the groups (*p* < 0.001). Error bars in the bar graph represent the mean with standard deviation (SD). The Kruskal–Wallis test was used to compare the differences among the groups. (**B**) ROC of average *TRH* methylation of five CpGs to differentiate between normal and AIN1+ lesions. AUC was 0.7674 (95% CI: 0.6544–0.8804) (*p*-value = 0.0006). (**C**) ROC of average *TRH* methylation of five CpGs to differentiate between ≤AIN1 and AIN2+ lesions. AUC was 0.7667 (95% CI: 0.6728–0.8606) (*p*-value = 0.0001). (**D**) ROC of average *TRH* methylation of five CpGs to differentiate between ≤AIN2 and AIN3 lesions. AUC was 0.8048 (95% CI: 0.6991–0.9106) (*p*-value = 0.0001).

Methylation analysis was performed by comparing between the normal, AIN1, AIN2 and AIN3 samples; the average *TRH* methylation levels of five CpGs of each group were 6.5%, 10.58%, 13.0%, and 25.65%, respectively (Table 1). There were statistically significant differences in average *TRH* methylation among the groups (*p* < 0.001) (Figure 3A).

ROC analysis was performed comparing average *TRH* methylation levels between two groups of three models, namely normal vs. AIN1-3, ≤AIN1 vs. AIN2+, and ≤AIN2 vs. AIN3; the areas under the ROC curve (AUC) were 0.7674, 0.7667, and 0.8048, respectively (Figure 3B–D). The sensitivity and specificity data obtained from ROC analysis are presented in Table 2 and Supplementary Table S3. The TRH methylation cut-off values considered optimal in the present study which have the acceptable sensitivity (>70%) and specificity (>50%) were 6.5% of normal vs. AIN1-3, 8.5% of ≤AIN1 vs. AIN2+, and 8.5% of ≤AIN2 vs. AIN3, as highlighted in Table 2. The AUC values, sensitivity and specificity for individual CpG are shown in Table 1 and Supplementary Table S3, respectively.

### 2.3. Diagnostic Performance of Cytology and TRH Methylation in Detecting for AIN1+, AIN2+ and AIN3 Lesions

The diagnostic performance of cytology and *TRH* methylation was calculated according to the histological results as a gold standard for AIN diagnosis. The diagnostic performance of cytology in predicting AIN1+ and AIN2+ lesions demonstrated a low sensitivity (25.37% and 19.23%, respectively) but a 100% specificity. The positive predictive value (PPV) was 100%, showing that most positive cytology (LSIL and HSIL) results correctly identified true positives (AIN1+ and AIN2+). The negative predictive value (NPV) and accuracy for predicting AIN2+ were higher than detecting AIN1+ (72% vs. 20.63% for NPV and 73.75% vs. 37.50% for accuracy) (Table 3).

The average of *TRH* methylation values of 6.5%, 8.5% and 8.5% was used to evaluate their diagnostic performance in detecting for AIN1+, AIN2+ and AIN3, respectively. The selected methylation cut-off values were applied to classify each sample as either “positive” (methylation above the cut-off) or “negative” (methylation below the cut-off). These binary classifications were compared with histological diagnoses using 2 × 2 contingency tables, from which sensitivity and specificity were calculated for each diagnostic model and cut-off point. The highest sensitivity was found in detecting AIN3, followed by AIN2+ and AIN1+. However, the accuracy in detecting AIN1+ was higher than detecting AIN2+ and AIN3. The results showed that the *TRH* methylation demonstrated greater sensitivity and accuracy than the cytology to predict abnormal anal cells. The *TRH* methylation cut-off value of 6.5% of the average *TRH* methylation also exhibited higher accuracy than the cytology for predicting AIN1+, improving from 37.50% to 73.33% (Table 3). To increase the specificity and PPV of the *TRH* methylation, the increased cut-off point was analyzed to evaluate the diagnostic performance; however, the sensitivity and NPV were decreased as shown in Table 4.

To improve the screening test for anal cancer, the combination of cytology and/or *TRH* methylation was evaluated. Positive cytology or *TRH* methylation results showed higher sensitivity (80.60% and 83.33%) and accuracy (75.00% and 65.00%) in detecting AIN1+ and AIN2+, respectively, as shown in Table 5.

### 2.4. TRH Methylation Is Not Associated with HIV Status and CD4 Levels

**Figure 4:**
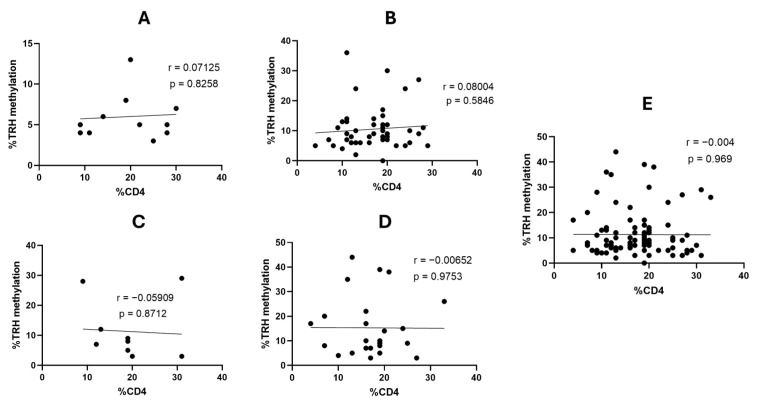
Correlation between the percentage of *TRH* methylation and the percentage of CD4 counts in patients with HIV with different AIN lesions. (**A**) Normal (12 samples), (**B**), AIN1 (49 samples), (**C**) AIN2 (10 samples), (**D**) AIN3 (25 samples) and (**E**) all 96 samples.

We further investigated whether HIV status and CD4 percentage have an effect on *TRH* methylation or not; it was found that *TRH* methylation levels at all five CG positions, as well as the average methylation across these CGs, gradually increased from normal to AIN3 (Table 6). There was no significant correlation with the percentage of CD4 in patients with HIV (*p* > 0.05 for all comparisons, Figure 4). However, the percentage of *TRH* gene methylation in patients with HIV increased significantly with disease progression, from normal to AIN3 lesions (*p* < 0.05).

## 3. Discussion

The present study focused on *TRH* methylation in the MSM group, particularly MSM-LHIV because it has a higher risk of developing anal cancer than in HIV-negative MSM [8,22]. In Thailand, the HIV prevalence among MSM was high, especially in Bangkok [23,24,25,26]. A high incidence of new HIV infections was observed among young MSM with age ≤ 22 years [27]. High-grade AIN was also highly detected in MSM with and without HIV [28]. Due to the anal cancer incidence being high in MSM and transgender women (TW) with HIV, the anal cancer screening for HIV-positive TW and MSM should begin around age 35. For MSM and TW who are HIV-negative and other HIV-positive individuals, screening should begin at age 45 [9]. The routine screening of anal cancer is anal cytology alone or co-testing (anal cytology and HPV testing) for early diagnosis of cancer and its precursor lesions. Individuals with abnormal results, such as ASCUS, LSIL with HR-HPV positive, and those with ASC-H or HSIL cytology, are referred to HRA, and biopsies should be collected [29]. However, the specificity of cytology and HPV testing was low [11,30]. HRA is considered the gold standard for diagnosing high-grade AIN and anal cancer, but it is invasive, time-consuming, requires expert skill, and is not feasible for resource-limited settings or low- to middle-income countries [10,11,12,31,32,33,34]. Molecular biomarkers, such as host DNA methylation, which reflects epigenetic changes occurring during the transition from normal tissue to precancerous and cancerous lesions, have been studied in many cancer types [35,36,37,38].

In this study, we demonstrated that the methylation levels of the *TRH* gene at cg01009664 in anal cells correlate with the progression of anal precancerous lesions that could be used to differentiate between normal and low-grade (AIN1-2) to high-grade (AIN3) lesions. *TRH* methylation may serve as a biomarker for predicting anal lesion severity. This is particularly important because high-grade lesions (AIN2+) have a higher risk of progressing to anal carcinoma [32,39,40,41,42] and identifying these at an earlier stage can reduce the incidence of anal cancer. The results of the present study is consistent with previous studies that *TRH* hypermethylation was detected in oral and oropharyngeal cancer and cervical cancer [16,17]. The present study had a high AUC of *TRH* methylation for predicting AIN2+ and AIN3+ lesions (0.7667 vs. 0.8048, respectively). The present study has shown that single *TRH* methylation has a high AUC for predicting AIN2+ and AIN3+.

In comparison, a meta-analysis of viral and host gene methylation found that the pooled AUC for the diagnosis of AIN2+ was 0.68 (0.63–0.73) [43], which is lower than the present study. Many studies reported that methylation panels could increase the AUC for predicting AIN3+ in HIV-positive groups, such as *LHX8* and *ZNF582* or *ASCL1* and *ZNF582* panels, which revealed AUCs of 0.70 and 0.69, respectively. The single-gene methylation marker had a low AUC range from 0.55 to 0.68 for identifying HSIL cases [44]. A marker panel of *FMN2* and *ASCL1* (AUC of 0.725) showed better diagnostic performance than *FMN2* or *ASCL1* alone (AUCs of 0.723 and 0.658, respectively) for prediction of HSIL (AIN2-3) [45]. A marker panel of *ASCL1* and *ZIC1* had an AUC of 0.85 for prediction of AIN3+ [46]. A five-marker panel including *ASCL1*, *ST6GALNAC3*, *WDR17*, *ZIC1*, and *ZNF582* had an AUC of 0.90 for prediction of AIN3+ [47]. Another marker panel consisting of *ASCL1*, *SST*, and *ZNF582* showed an AUC of 0.89 [48]. The present study has shown that single TRH methylation has a high AUC for predicting AIN2+ and AIN3+. TRH represents a single-gene methylation marker that may provide a simpler, more cost-effective, and more accessible assay while maintaining diagnostic performance that is comparable to or better than other panel tests mentioned above. Importantly, TRH hypermethylation has been consistently observed in many HPV-related cancers, including oral and cervical cancer [16,17].

Our study showed that there was no correlation between CD4 counts and *TRH* methylation in any anal lesions; however, it was found at CG position 5 showed moderate correlation between AIN3 and CD4 percentage (r = 0.6372, Supplementary Figure S1). One study reported that there was moderate correlation between some CG positions of HPV16 L1 gene and CD4 count in high-grade AIN HIV-positive patients [49].

The elevated methylation levels in high-grade anal lesions indicated its association with the progression of dysplastic and neoplastic lesions as shown by a previous study that *endothelin 3 (EDN3)* silencing by methylation promotes cervical cancer cell proliferation and invasion [50]. The gradual increase in methylation from normal to AIN1-2 and AIN3 highlights the potential of the *TRH* gene as a dynamic marker that reflects the severity of cellular abnormalities in the anal cells. Further research is needed to explore the role of *TRH* methylation in AIN progression and its potential interaction with other molecular pathways involved in anal carcinogenesis.

However, this study has limitations, including small sample sizes, which may weaken the strength of the findings. More research with larger sample sizes is required to confirm that the findings may be used in clinical settings for distinguishing normal cells from AIN and cancer. In conclusion, our study highlights the potential of *TRH* methylation as a biomarker for identifying and differentiating AIN lesions, indicating the possibility of enhanced diagnostics in anal neoplasia management. As a result, *TRH* methylation may help to reduce the need for immediate referral to HRA or to help clinicians in deciding whether patients should be referred for HRA or not.

## 4. Materials and Methods

### 4.1. Clinical Samples and Cell Lines

The present study is a retrospective study of 120 archived DNA samples extracted from anal cells including 16 negatives for malignancy, 66 AIN I, 12 AIN II and 26 AIN III. There were 96 HIV-positive samples and 24 HIV-negative samples. These samples were collected from MSM at the Thai Red Cross AIDS Research Centre (TRC-ARC), Bangkok, Thailand, during May to December 2013. All of the samples were anonymized and bisulfite-treated DNA samples were leftovers from a previous study [49]; therefore, informed consent was not required. The DNA extracted from human cervical cancer cell lines containing HPV16, i.e., CaSki (ATCC CRL-1550, Manassas, VA, USA) and SiHa (ATCC HTB-35, Manassas, VA, USA), human cervical cancer cell line without HPV; C33A (ATCC HTB-31, Manassas, VA, USA), and normal human cervical epithelial cells, PCS480011 (ATCC PCS-480-011™, Manassas, VA, USA) were used as a control for hypermethylated and hypomethylated *TRH* gene. The DNA samples were used for bisulfite treatment using the EZ DNA Methylation-Gold kit (Zymo Research, Irvine, CA, USA).

We retrieved methylation profiling by genome tiling array GSE186859 data by accessing GENBANK on 30 January 2024 (http://www.ncbi.nlm.nih.gov/geo, the data was available in NCBI on 29 November 2021), using the GPL13534 Illumina HumanMethylation450 BeadChip platform (HumanMethylation450_15017482, Illumina Inc., San Diego, CA, USA). There were 143 FFPE anal samples including 121 invasive tumors, 13 adjacent AIN3 and 9 adjacent normal [21]. This study was approved by the Institutional Review Board of the Faculty of Medicine, Chulalongkorn University (COA No. 0191/2025, IRB No. 0043/68, Date of approval: 6 February 2025), and the Institutional Biosafety Committee (IBC) of the Faculty of Medicine, Chulalongkorn University (MDCU-IBC001/2025, Effective date: 17 February 2025).

### 4.2. Polymerase Chain Reaction of Bisulfite-Treated DNA

The EZ DNA Methylation-Gold kit (Zymo Research, Irvine, CA, USA) was used to bisulfite treatment following the manufacturer’s procedure. The sequences of forward, reverse, and sequencing primers were as follows: Forward Primer—5′-GGGGTTTTTAGAGTTGTAGATTTTTGA-3′; Reverse Primer—Biotin 5′-CCAAAAATAAACTCCACAAAATAAATC-3′. The *TRH* sequencing primer was 5′-TTTAGAGTTGTAGATTTTTGATTTG-3′ and the sequence to analyze was TYGATTGYGGATTTYGAGTTTTYGGATTTYGGATTTATTTTGT; bisulfite-treated DNA in each sample was used for PCR using TaKaRa EpiTaq™ HS (Cat. #R110A, Takara Bio, Shiga, Japan). Reagent concentrations per 25 µL reaction tube were as follows: 1X of 10XEpiTaq PCR Buffer (Mg^2+^ free), 2.5 mM MgCl_2_, 0.3 mM dNTPs (2.5 mM each), 0.8 µM each primer, 0.625 U Taq polymerase (TaKaRa EpiTaq HS), 2 µL of bisulfite-treated DNA each sample and DNase/RNase-free water were added to the final volume of 25 µL. The PCR amplification was started with an initial denaturing at 95 °C for 2 min, followed by 45 cycles of 98 °C for 10 s, 53 °C for 1 min, and 72 °C for 1 min, and a cycle for the final extension at 72 °C for 5 min. The PCR products were detected by 1.5% agarose gel electrophoresis.

### 4.3. Methylation Analysis by a Pyrosequencing Assay

Pyrosequencing was performed using the PSQ96MA System (Biotage, Uppsala, Sweden) according to the manufacturer’s protocol. Briefly, prior to pyrosequencing, 20 µL of biotin-labeled amplified products were mixed with beads, washed with 70% ethanol, denatured, washed and mixed with 0.4 µM of sequencing primers and loaded into the PyroMark™ Q96 machine (Qiagen, Hilden, Germany). Following the run, the program will calculate each CpG site’s percentage of methylation level, as shown in the pyrogram analysis. The bisulfite conversion control, which indicates that a single cytosine is entirely converted to uracil, is shown by a yellow bar. The sequence’s investigated CpG sites are shown by the gray bar. On top of the gray bar, each CpG site that passes the quality check has its percentage of methylation level shown in blue.

### 4.4. Receiver Operating Characteristic (ROC) Curve Analysis

A receiver operating characteristic (ROC) curve analysis was performed to evaluate the diagnostic performance of *TRH* methylation across three clinically relevant comparison models: (1) Normal vs. AIN1–3 represents a primary screening distinction between lesion-free individuals and those with any grade of AIN (2) ≤AIN1 vs. AIN2+ is a triage threshold used to identify individuals who may require referral for high-resolution anoscopy, as AIN2+ represents high-grade disease; and (3) ≤AIN2 vs. AIN3 differentiates the most advanced precancerous lesions with the greatest likelihood of progression. Average methylation percentages of each sample with different anal lesions obtained from pyrosequencing were analyzed as continuous variables. ROC curves, the area under the curve (AUC) with corresponding 95% confidence intervals and sensitivity and specificity were generated using GraphPad Prism 10.0.3 (GraphPad Software, Inc., San Diego, CA, USA).

To further characterize diagnostic accuracy, the selected methylation cut-off values that showed the best sensitivity and specificity derived from the ROC curves were applied to each comparison model. For each cut-off, TRH methylation values above the cut-off value were classified as positive, and values below the cut-off value were classified as negative. The diagnostic performance of TRH methylation was also compared with cytology using histology as the reference standard. These binary classifications were compared with histological diagnoses using 2 × 2 contingency tables. All diagnostic indices were calculated using the MedCalc Diagnostic Test Calculator (MedCalc Software Ltd., Ostend, Belgium; available online at https://www.medcalc.org/en/calc/diagnostic_test.php; accessed on 6 February 2025).

### 4.5. Statistical Analysis

Statistical analysis was performed using GraphPad Prism 10.0.3 (GraphPad Software, Inc., San Diego, CA, USA). All information was analyzed as a mean and percentage. A receiver operating characteristic (ROC) curve was constructed to evaluate the diagnostic capability of *TRH* methylation levels in distinguishing between normal and AIN1+. The Kruskal–Wallis test was used to examine the differences in the mean percentage of methylation levels among groups. Pearson’s correlation coefficient (r) was used to analyze the association between the percentage of methylation and the CD4 and %CD4 count. A statistically significant difference was defined as a *p*-value of less than 0.05.

## Figures and Tables

**Table 1:** The percentage of *TRH* methylation of each CG using pyrosequencing.

Anal Cell Lesions	CG1 (%Mean)	CG2 (%Mean)	CG3 (%Mean)	CG4 (%Mean)	CG5 (%Mean)	CG Average (%Mean)
Normal (N = 16)	9.12	7.56	6.25	5.31	2.94	6.25
AIN1 (N = 66)	13.53	12.91	12.11	8.53	5.58	10.58
AIN2 (N = 12)	14.50	16.75	17.33	9.83	6.50	13.00
AIN3 (N = 26)	25.69	25.92	28.73	26.15	22.04	25.65
Total (N = 120)	15.68	15.40	15.45	12.05	8.88	13.50
AUC Normal vs. AIN1-3 (95% confidence interval)	0.73 (0.59 to 0.87)	0.77 (0.66 to 0.87)	0.77 (0.67 to 0.87)	0.70 (0.58 to 0.81)	0.70 (0.58 to 0.80)	0.77 (0.65 to 0.88)
AUC ≤AIN1 and AIN2+ (95% confidence interval)	0.69 (0.58 to 0.79)	0.75 (0.64 to 0.84)	0.78 (0.69 to 0.86)	0.78 (0.68 to 0.87)	0.78 (0.69 to 0.87)	0.77 (0.67 to 0.86)
AUC ≤AIN2 and AIN3 (95% confidence interval)	0.75 (0.63 to 0.87)	0.77 (0.65 to 0.88)	0.79 (0.68 to 0.89)	0.84 (0.74 to 0.92)	0.86 (0.76 to 0.94)	0.80 (0.69–0.91)

**Table 2:** Sensitivity and specificity of *TRH* methylation at different cut-off points obtained from ROC analysis to compare average *TRH* methylation levels of five CpGs of three models, namely normal and AIN1-3, ≤AIN1 and AIN2+ and ≤AIN2 and AIN3.

Average *TRH* Methylation (Normal vs. AIN1+)					Average *TRH* Methylation (≤AIN1 vs. AIN2+)					Average *TRH* Methylation (≤AIN2 vs. AIN3)				
Cut-Off (%)	Sensitivity %	95% CI	Specificity %	95% CI	Cut-Off (%)	Sensitivity %	95% CI	Specificity %	95% CI	Cut-Off (%)	Sensitivity %	95% CI	Specificity %	95% CI
>1.00	99.04	94.75% to 99.95%	6.25	0.32% to 28.33%	>1.00	100	90.82% to 100.0%	2.43	0.43% to 8.46%	>1.00	100	87.13% to 100.0%	2.128	0.37% to 7.42%
>2.50	97.12	91.86% to 99.21%	6.25	0.32% to 28.33%	>2.50	100	90.82% to 100.0%	4.87	1.91% to 11.88%	>2.50	100	87.13% to 100.0%	4.255	1.66% to 10.44%
>3.50	97.12	91.86% to 99.21%	12.5	2.22% to 36.02%	>3.50	100	90.82% to 100.0%	6.09	2.63% to 13.49%	>3.50	100	87.13% to 100.0%	5.319	2.29% to 11.85%
>4.50	95.19	89.24% to 97.93%	31.25	14.16% to 55.60%	>4.50	97.37	86.51% to 99.87%	10.98	5.88% to 19.56%	>4.50	96.15	81.11% to 99.80%	9.574	5.11% to 17.20%
>5.50	86.54	78.66% to 91.81%	50	28.00% to 72.00%	>5.50	94.74	82.71% to 99.06%	24.39	16.38% to 34.69%	>5.50	96.15	81.11% to 99.80%	22.34	15.10% to 31.75%
>6.50	75.96	66.92% to 83.15%	56.25	33.18% to 76.90%	>6.50	89.47	75.87% to 95.83%	36.59	26.98% to 47.39%	>6.50	92.31	75.86% to 98.63%	34.04	25.26% to 44.08%
>7.50	65.38	55.84% to 73.83%	62.5	38.64% to 81.52%	>7.50	81.58	66.58% to 90.78%	47.56	37.10% to 58.24%	>7.50	84.62	66.47% to 93.85%	44.68	35.04% to 54.74%
>8.50	58.65	49.05% to 67.65%	81.25	56.99% to 93.41%	>8.50	78.95	63.65% to 88.93%	58.54	47.73% to 68.58%	>8.50	84.62	66.47% to 93.85%	55.32	45.26% to 64.96%
>9.50	50	40.56% to 59.44%	87.5	63.98% to 97.78%	>9.50	71.05	55.24% to 83.00%	67.07	56.34% to 76.28%	>9.50	76.92	57.95% to 88.97%	63.83	53.75% to 72.82%
>10.50	44.23	35.06% to 53.81%	87.5	63.98% to 97.78%	>10.50	63.16	47.28% to 76.62%	70.73	60.13% to 79.47%	>10.50	69.23	50.01% to 83.50%	68.09	58.11% to 76.64%
>11.50	41.35	32.35% to 50.95%	93.75	71.67% to 99.68%	>11.50	63.16	47.28% to 76.62%	75.61	65.31% to 83.62%	>11.50	69.23	50.01% to 83.50%	72.34	62.56% to 80.37%
>12.50	37.5	28.80% to 47.09%	93.75	71.67% to 99.68%	>12.50	60.53	44.72% to 74.40%	79.27	69.28% to 86.63%	>12.50	69.23	50.01% to 83.50%	76.6	67.10% to 84.01%
>13.50	32.69	24.43% to 42.18%	100	80.64% to 100.0%	>13.50	55.26	39.71% to 69.85%	84.15	74.74% to 90.49%	>13.50	69.23	50.01% to 83.50%	82.98	74.13% to 89.24%
>14.50	29.81	21.86% to 39.19%	100	80.64% to 100.0%	>14.50	52.63	37.26% to 67.52%	86.59	77.55% to 92.34%	>14.50	65.38	46.22% to 80.59%	85.11	76.54% to 90.92%

Gray-Highlighted values represent *TRH* methylation cut-off points selected based on optimal sensitivity and specificity from ROC analysis.

**Table 3:** Sensitivity, specificity, positive predictive value, (PPV), negative predictive value (NPV) and accuracy of each test to predict AIN1+, AIN2+ and AIN3 lesions.

Results of Pap Smear to Predict AIN1+ (Exclude ASCUS)		Histology			Parameter	Point Estimates (95% CI)
		AIN1+	Normal	Total	Sensitivity	25.37% (15.53% to 37.49%)
Cytology	LSIL/HSIL	17	0	17	Specificity	100.00% (75.29% to 100.00%)
	Normal	50	13	63	PPV	100.00% (80.49% to 100.00%)
	Total	67	13	80	NPV	20.63% (18.44% to 23.02%)
					Accuracy	37.50% (26.92% to 49.04%)
Results of Pap smear to predict AIN2+ (exclude ASCUS)		Histology			Parameter	Point Estimates (95% CI)
		AIN2+	≤AIN1	Total	Sensitivity	19.23% (6.55% to 39.35%)
Cytology	HSIL	5	0	5	Specificity	100.00% (93.40% to 100.00%)
	Normal/LSIL	21	54	75	PPV	100.00% (47.82% to 100.00%)
	Total	26	54	80	NPV	72.00% (68.07% to 75.62%)
					Accuracy	73.75% (62.71% to 82.96%)
Results of average *TRH* methylation levels to predict AIN1+		Histology			Parameter	Point Estimates (95% CI)
		AIN1+	Normal	Total	Sensitivity	75.96% (66.59% to 83.80%)
average *TRH* methylation levels	≥6.50%	79	7	86	Specificity	56.25% (29.88% to 80.25%)
	<6.50%	25	9	34	PPV	91.86% (86.50% to 95.21%)
		104	16	120	NPV	26.47% (17.19% to 38.44%)
					Accuracy	73.33% (64.49% to 80.99%)
Results of average *TRH* methylation levels to predict AIN2+		Histology			Parameter	Point Estimates (95% CI)
		AIN2+	≤AIN1	Total	Sensitivity	78.95% (62.68% to 90.45%)
average *TRH* methylation levels	≥8.50%	30	34	64	Specificity	58.54% (47.12% to 69.32%)
	<8.50%	8	48	56	PPV	46.87% (39.41% to 54.49%)
		38	82	120	NPV	85.71% (75.94% to 91.94%)
					Accuracy	65.00% (55.76% to 73.48%)
Results of average *TRH* methylation levels to predict AIN3		Histology			Parameter	Point Estimates (95% CI)
		AIN3	≤AIN2	Total	Sensitivity	84.62% (65.13% to 95.64%)
average *TRH* methylation levels	≥8.50%	22	42	64	Specificity	55.32% (44.71% to 65.59%)
	<8.50%	4	52	56	PPV	34.38% (28.40% to 40.90%)
		26	94	120	NPV	92.86% (83.83% to 97.02%)
					Accuracy	61.67% (52.35% to 70.39%)

**Table 4:** Sensitivity, specificity, positive predictive value, (PPV), negative predictive value (NPV) and accuracy of TRH methylation at different cut-off points to predict AIN1+, AIN2+ and AIN3 lesions.

	Cut-Off	6.5%	8.5%	10%	20%
Comparison	Parameter	Values (95% CI)	Values (95% CI)	Values (95% CI)	Values (95% CI)
Average *TRH* methylation levels to predict AIN1+	Sensitivity	75.96% (66.59% to 83.80%)	58.00% (47.71% to 67.08%)	50.00% (40.03% to 59.97%)	25.00% (17.03% to 34.45%)
	Specificity	56.25% (29.88% to 80.25%)	78.57% (49.20% to 95.34%)	87.50% (61.65% to 98.45%)	100.00% (79.41% to 100.00%)
	PPV	91.86% (86.50% to 95.21%)	95.08% (87.49% to 98.16%)	96.30% (87.52% to 98.97%)	100.00% (86.77% to 100.00%)
	NPV	26.47% (17.19% to 38.44%)	20.75% (15.48% to 27.25%)	21.21% (17.09% to 26.01%)	17.02% (15.51% to 18.65%)
	Accuracy	73.33% (64.49% to 80.99%)	60.53% (50.94% to 69.55%)	55.00% (45.65% to 64.09%)	35.00% (26.52% to 44.24%)
	Cut-off	6.5%	8.5%	10%	20%
Comparison	Parameter	Values (95% CI)	Values (95% CI)	Values (95% CI)	Values (95% CI)
Average *TRH* methylation levels to predict AIN2+	Sensitivity	89.47% (75.20% to 97.06%)	78.95% (62.68% to 90.45%)	71.05% (54.10% to 84.58%)	50.00% (33.38% to 66.62%)
	Specificity	36.59% (26.22% to 47.95%)	58.54% (47.12% to 69.32%)	67.07% (55.81% to 77.06%)	91.46% (83.20% to 96.50%)
	PPV	39.53% (34.93% to 44.33%)	46.87% (39.41% to 54.49%)	50.00% (40.86% to 59.14%)	73.08% (55.53% to 85.51%)
	NPV	88.24% (73.98% to 95.19%)	85.71% (75.94% to 91.94%)	83.33% (74.81% to 89.38%)	79.79% (74.04% to 84.52%)
	Accuracy	53.33% (44.01% to 62.49%)	65.00% (55.76% to 73.48%)	68.33% (59.22% to 76.52%)	78.33% (69.89% to 85.33%)
	Cut-off	6.5%	8.5%	10%	20%
Comparison	Parameter	Values (95% CI)	Values (95% CI)	Values (95% CI)	Values (95% CI)
Average TRH methylation levels to predict AIN3	Sensitivity	92.31% (74.87% to 99.05%)	84.62% (65.13% to 95.64%)	76.92% (56.35% to 91.03%)	61.54% (40.57% to 79.77%)
	Specificity	34.04% (24.58% to 44.54%)	55.32% (44.71% to 65.59%)	63.83% (53.27% to 73.49%)	89.36% (81.30% to 94.78%)
	PPV	27.91% (24.38% to 31.73%)	34.38% (28.40% to 40.90%)	37.04% (29.49% to 45.28%)	61.54% (45.26% to 75.58%)
	NPV	94.12% (80.40% to 98.42%)	92.86% (83.83% to 97.02%)	90.91% (82.98% to 95.35%)	89.36% (83.71% to 93.21%)
	Accuracy	46.67% (37.51% to 55.99%)	61.67% (52.35% to 70.39%)	66.67% (57.48% to 75.01%)	83.33% (75.44% to 89.51%)

**Table 5:** Sensitivity, specificity, positive predictive value, (PPV), negative predictive value (NPV) and accuracy of each combined test to predict AIN1+ and AIN2+ lesions.

Combined Pap Smear AND Methylation to Predict AIN1+ (Exclude ASCUS)		Histology			Parameter	Point Estimates (95% CI)
		AIN1+	Normal	Total		
Cytology	≥6.50%/LSIL/HSIL	13	7	20	Sensitivity	19.40% (10.76% to 30.89%)
					Specificity	46.15% (19.22% to 74.87%)
	<6.50%/Normal/LSIL/HISL ≥6.50%/Normal	54	6	60	PPV	65.00% (47.95% to 78.92%)
					NPV	10.00% (5.75% to 16.82%)
	Total	67	13	80	Accuracy	23.75% (14.95% to 34.58%)
Combined Pap smear AND methylation to predict AIN2+ (exclude ASCUS)		Histology			Parameter	Point Estimates (95% CI)
		AIN2+	≤AIN1	Total		
Cytology	≥8.50%/HSIL	3	0	3	Sensitivity	12.50% (2.66% to 32.36%)
					Specificity	100.00% (93.62% to 100.00%)
	<8.50%/Normal/LSIL/HSIL ≥8.50%/Normal/LSIL	21	56	77	PPV	100.00% (29.24% to 100.00%)
					NPV	72.73% (69.63% to 75.62%)
	Total	24	56	80	Accuracy	73.75% (62.71% to 82.96%)
Combined Pap smear OR methylation to predict AIN1+ (exclude ASCUS)		Histology			Parameter	Point Estimates (95% CI)
		AIN1+	Normal	Total		
Cytology	≥6.50%or Normal/LSIL/HSIL <6.50% or LSIL/HSIL	54	7	61	Sensitivity	80.60% (69.11% to 89.24%)
					Specificity	46.15% (19.22% to 74.87%)
	<6.50% or Normal	13	6	19	PPV	88.52% (82.15% to 92.82%)
					NPV	31.58% (17.70% to 49.76%)
	Total	67	13	80	Accuracy	75.00% (64.06% to 84.01%)
Combined Pap smear OR methylation to predict AIN2+ (exclude ASCUS)		Histology			Parameter	Point Estimates (95% CI)
		AIN2+	≤AIN1	Total		
Cytology	≥8.50%or Normal/LSIL/HSIL <8.50% or HSIL	20	24	44	Sensitivity	83.33% (62.62% to 95.26%)
					Specificity	57.14% (43.22% to 70.29%)
	<8.50% or Normal/LSIL	4	32	36	PPV	45.45% (36.96% to 54.22%)
					NPV	88.89% (76.07% to 95.27%)
	Total	24	56	80	Accuracy	65.00% (53.52% to 75.33%)

**Table 6:** Mean *TRH* methylation level of each CpG and the percentage of CD4 count between patients with HIV and patients without HIV.

Group	Normal (16)		AIN1(66)		AIN2(12)		AIN3(26)		*p*-Value
HIV	Without HIV	With HIV	Without HIV	With HIV	Without HIV	With HIV	Without HIV	With HIV	
NO. (120)	4	12	17	49	2	10	1	25	
Mean %CD4	NA	19.50%	NA	16.84%	NA	19.20%	NA	17.04%	0.273
Mean cell count	NA	388.83	NA	315.16	NA	358.80	NA	319.68	0.352
Mean % *TRH* methylation									
CpG 1	9.00%	9.17%	13.76%	13.45%	28.50%	11.70%	5.00%	26.52%	<0.001
CpG 2	9.75%	6.83%	13.18%	12.82%	30.50%	14.00%	6.00%	26.72%	<0.001
CpG 3	8.25%	5.58%	10.53%	12.65%	29.00%	15.00%	5.00%	29.68%	<0.001
CpG 4	5.25%	5.33%	8.88%	8.41%	20.50%	7.70%	8.00%	26.88%	<0.001
CpG 5	3.00%	2.92%	6.82%	5.14%	15.00%	4.80%	5.00%	22.72%	<0.001
Average CG1-5	7.00%	6.00%	10.71%	10.53%	24.50%	10.70%	6.00%	26.44%	<0.001

## Data Availability

The raw data used in this research would be available from the corresponding author upon reasonable request.

## Supplementary Materials

The following supporting information can be downloaded at https://www.mdpi.com/article/10.3390/ijms262411784/s1.

## Abbreviations

AIN	Anal intraepithelial neoplasia
ASC-H	Atypical squamous cells, cannot exclude HSIL
ASC-US	Atypical squamous cells of undetermined significance
AUC	Area under the curve
HRA	High-resolution anoscopy
HSIL	High-grade squamous intraepithelial lesion
KEGG	Kyoto Encyclopedia of Genes and Genomes
LSIL	Low-grade squamous intraepithelial lesion
MSM-LWHIV	Men who have sex with men living with HIV
NPV	Negative predictive value
PPV	Positive predictive value
ROC	Receiver operating characteristic
TRH	Thyrotropin-releasing hormone
