# Supplementary material for: Thyrotropin-Releasing Hormone Gene Methylation as a Potential Biomarker for Anal Intraepithelial Neoplasia
Source: Int J Mol Sci. 2025 Dec 5;26(24):11784. doi: 10.3390/ijms262411784 (PMC12733343; doi:10.3390/ijms262411784)
Supplement: Supplementary file 1 [file ijms-26-11784-s001.zip › ijms-3963136-supplementary/Supplementary Figure S1 + figure legend.pdf]

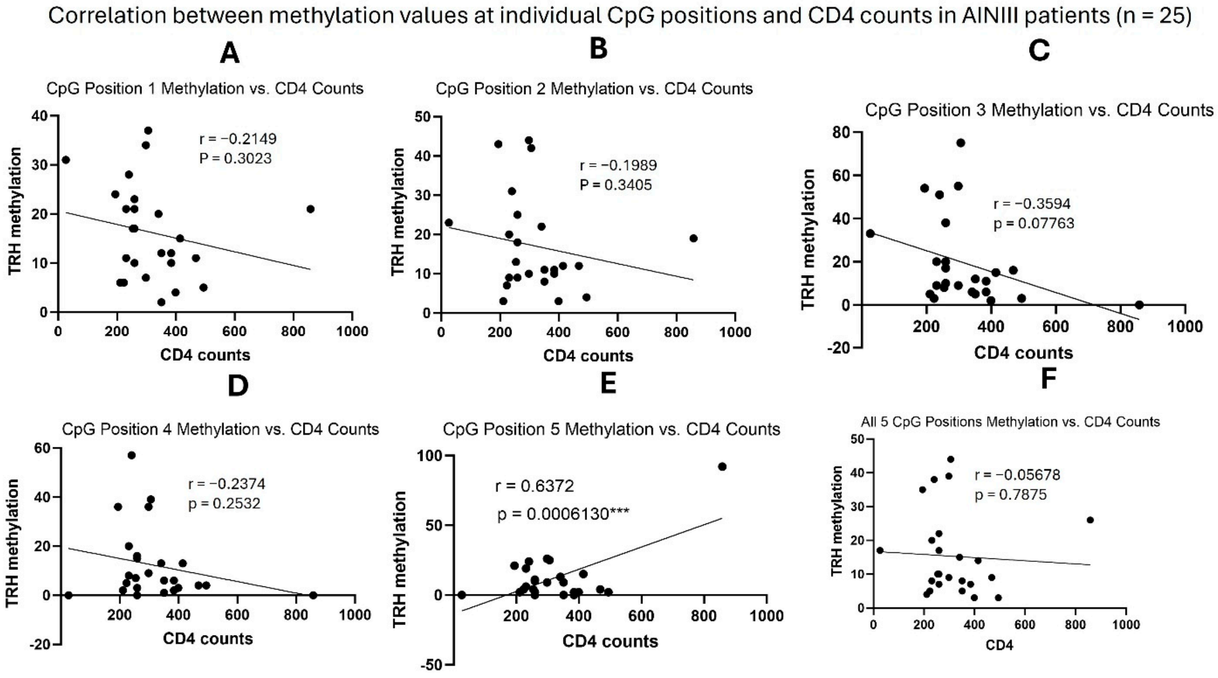

Supplementary Figure S1. Correlation between the percentage of TRH methylation and the percentage of CD4 counts in patients with HIV with AIN III lesions. (A) CpG position 1, (B), CpG position 2, (C) CpG position 3, (D) CpG position 4, (E) CpG position 5 and (F) All 5 CpGs.
